# Antifungal Nanoformulation for Biocontrol of Tomato Root and Crown Rot Caused by *Fusarium oxysporum* f. sp. *radicis*-*lycopersici*

**DOI:** 10.3390/antibiotics10091132

**Published:** 2021-09-20

**Authors:** Ricardo Aravena, Ximena Besoain, Natalia Riquelme, Aldo Salinas, Miryam Valenzuela, Eduardo Oyanedel, Wilson Barros, Yusser Olguin, Alejandro Madrid, Matias Alvear, Iván Montenegro

**Affiliations:** 1 Escuela de Agronomia, Facultad de Ciencias Agronomicas y de los Alimentos, Pontificia Universidad Catolica de Valparaiso, San Francisco s/n La Palma, Quillota 2260000, Chile; ricardo.aravenaflores@gmail.com (R.A.); natalia.riquelme@pucv.cl (N.R.); aldo.salinas@pucv.cl (A.S.); eduardo.oyanedel@pucv.cl (E.O.); wilson.barros@pucv.cl (W.B.); 2 Molecular Microbiology and Environmental Biotechnology Laboratory, Department of Chemistry & Center of Biotechnology Dr. Daniel Alkalay Lowitt, Universidad Tecnica Federico Santa Maria, Avda. España 1680, Valparaiso 2390123, Chile; miryam.valenzuela@sansano.usm.cl; 3 Centro Cientifico Tecnologico de Valparaiso—CCTVaL, Universidad Tecnica Federico Santa Maria, Avenida España 1680, Valparaiso 2340000, Chile; yusser.olguin@usm.cl; 4 Laboratorio de Productos Naturales y Sintesis Organica—LPNSO, Departamento de Quimica, Facultad de Ciencias Naturales y Exactas, Universidad de Playa Ancha, Avda. Leopoldo Carvallo 270, Playa Ancha, Valparaiso 2340000, Chile; alejandro.madrid@upla.cl; 5 Laboratory of Industrial Chemistry, Process Chemistry Centre, Abo Akademi University, Biskopsgatan 8, 20500 Turku/Abo, Finland; matias.alvear@abo.fi; 6 Escuela de Obstetricia y Puericultura, Facultad de Medicina, Universidad de Valparaiso, Angamos 655, Reñaca, Vina del Mar 2520000, Chile

**Keywords:** *Fusarium oxysporum*, crown and root rot, plant extract, nanoemulsions, tomato plants

## Abstract

Tomatoes (*Solanum lycopersicum* L.) are the most cultivated and important vegetable crop in the world. These plants can wilt during crop growth due to fusarium wilt (fusariosis), a disease that damages tomato vascular systems. The *Fusarium* isolated and analyzed in this work correspond to *Fusarium oxysporum* f. sp. *radicis-lycopersici*. The isolates were molecularly identified, and analysis was done on the in vitro effects of the nanoemulsions (previously obtained from extracts of Chilean medicinal plants of the genera *Psoralea* and *Escallonia*) to inhibit mycelial and conidial germination of the isolates. Subsequently, the nanoemulsions were evaluated under greenhouse conditions for preventive control of fusariosis in the root and crown, with high levels of disease control observed using the highest concentrations of these nanoemulsions, at 250 and 500 ppm.

## 1. Introduction

The tomato (*Solanum lycopersicum* L.) is considered the most important and widely cultivated vegetable crop worldwide. Globally, production reached 4.7 million hectares (ha), equivalent to 177 million tons (t) of tomatoes in 2016 [1] with China, India and the United States being the largest producers [2]. In Chile, it is the most widely consumed vegetable, and approximately 5463 hectares are cultivated yearly for fresh tomato production [1].

One of the major production problems for growing tomato crops is fusarium wilt disease, or fusariosis, caused by the fungus *Fusarium oxysporum* f. sp. *lycopersici* (*Fol*) or by *F. oxysporum* f. sp. *radicis-lycopersici* (*Forl*). Both are morphologically indistinguishable, which can make their identification difficult [3,4]. These pathogens mainly infect plants by entering through the root elongation zone and through micro-wounds caused by root growth [5,6,7]. The availability of water facilitates entry and infection, causing symptoms of wilting from the basal zone to the apex of the plants by *Fol*, and root and crown rot by *Forl* [4]. The latter can also be found in asymptomatic host plants of the families: Amaranthaceae, Asteraceae, Brassicaceae, Chenopodiaceae and Poaceae [8].

From the roots, *Fol* moves acropetally through the xylem until it reaches the apex, where it blocks the vessels after having produced enzymes and toxins [9], whereas *Forl* begins by colonizing the areas where roots are growing, then moving towards the crown of the plant, and staying in that area [10]. Brown vascular lesions can be observed near the crowns of affected plants [5]. As is common in soil microorganisms, they can survive almost indefinitely in the form of chlamydospores (resistant survival structures). These pathogens can also be found in the soil as mycelium and colonizing the remains of the roots of host plants [11]. They can also be spread by tools, infected seeds, potting materials, contaminated soil, irrigation water, wind and insects [12]. *Fol* can cause yield losses of 21 to 47% [13], and *Forl*, losses of up to 78% of plants when they are grown in coconut fiber [14], since infected plants must be completely uprooted to prevent the pathogen from entering nearby plants [15]. 

*Fusarium* is a complex of fungal species. There are four forms which have been found to be especially damaging to tomato cultivation: *F. o.* f. sp. *lycopersici* races 1, 2 and 3 (*Fol*: 1, 2 and 3), and *F. o.* f. sp. *radicis-lycopersici* (Forl), with race 3 and Forl causing the greatest damage [16]. The majority of commercial hybrid tomato varieties have resistance to *Fol* 1 and 2, but not *Fol* 3 nor *Forl*, leaving plants without resistance to these two particular forms [17]. 

Currently, there are no adequate curative controls, leaving preventive control as the most common method of control. However, this has not been effective. Preventive control methods include soil solarization, vaporization, and the use of fumigants such as dazomet and metham sodium, among others. Growers also frequently burn crop residues, use resistant cultivars and crop rotation, as well as chemical controls [18]. 

In addition to the preventive soil fumigants such as metham sodium, fungicides such as carbendazim, benomyl, and prochloraz are also used to try to control *Fusarium*. It has been observed that the fungicides currently being applied are not very effective in controlling *Fusarium*, which could be due to structural resistance or its ability to infect alternative host plants [11,16]. Also, the use of chemical fungicides and fumigants should be increasingly limited due to current concerns about their impacts on the environment, health and global food safety [19]. The control of both of these specific pathogens depends mainly on chemicals [20], even when reports have been made showing that they are not effective once the pathogens have entered the plant [8]. The use of resistant rootstocks has also been implemented to prevent the development of *Fol* race 3 [21].

Alternatively, biological control methods, integrated with cultural practices, have been shown to reduce *Fusarium* incidence and severity and to help in the prevention of this disease [18]. The use of cyanobacteria for biological control has been shown to decrease wilting produced by Fol [22]. Fungi such as *Entrophospora colombiana*, *Trichoderma* spp., as well as the bacterium *Pseudomonas fluorescens* have also been tested as possible controls [23]. The combined use of *Trichoderma* spp. plus biofumigation with *Brassica juncea*, could have synergistic effects on control of the pathogen [18]. 

Another alternative to chemical control is the application of plant extracts. Extracts from the Chilean Canelo (*Drimys winteri* Forst) tree have been tested for effects on mycelial reduction of the pathogen compared to conventional controls [9,24]. Plant extracts from *Nicotiana tabacum* and *Chrisanthemus* have demonstrated antifungal activity without causing damage or adverse effects on growing plants [9,25]. Certain components of these plant extracts, such as cinnemaldehyde, eugenol, allyl isothiocyanate and carvacrol have also demonstrated an inhibitory capacity against the germination and vegetative growth of spores [26]. 

Applications of different plant extracts can inhibit mycelial growth of *Fusarium oxysporum*, including at significant levels, equal to conventional chemical controls [27]. These formulations can be very simple mixtures of natural ingredients with specific activity or more complex mixtures. The activity of these extracts refers to their ability to act as biofungicides, as this is the major benefit provided to the plants [28].

Mycelial inhibition studies done in vitro using commonly found plant extracts have demonstrated inhibition in mycelia of *Fusarium* spp., *Aspergillus flavus*, and *Rhizoctonia solani*, among others, with results equal to those obtained with benomyl and surpassing both the biological controls *Trichoderma* and *Bacillus*. The secondary metabolites that have been identified as contributors to the inhibitory action are either anthraquinonic in nature, or flavonoids of a catechinic nature [29,30].

At present, scientific studies on vegetable crops are moving towards nanotechnology, as the safest option for agrochemicals [31], exploring their use as antifungal agents [32] which in some cases have shown up to 100% inhibition of mycelial growth [33], with the same effects on *Fusarium oxysporum* conidia [34]. Nanoemulsions have been tested against *Fol*, revealing inhibition of this pathogen [35]. Are extracts based on Chilean medicinal plants effective for creating biopesticides that can defend tomatoes against this pathogen? Our hypothesis is that nanoemulsion formulations based on extracts from plants in the *Psoralea* and *Escallonia* genera will have a controlling effect on fusariosis in tomato plants.

To test this hypothesis, the following objectives were planned: (i) to evaluate the inhibitory effect of different plant extracts on the control of conidia in vitro for two isolates of *Fusarium oxysporum* f. sp. *radicis-lycopersici*, (ii) to evaluate the effect of nanoemulsions based on plant extracts to control fusariosis caused by *F. oxysporum* f. sp. *radicis-lycopersici*, when applied to tomato plants grown in a greenhouse. 

## 2. Materials and Methods

### 2.1. Obtaining and Identifying the Isolates

The isolates used in this study were obtained from monosporic cultures from the Phytopathology Laboratory (LF) of the Pontificia Universidad Católica de Valparaíso (PUCV), in La Palma, Quillota, Región de Valparaíso, Chile. Isolates were recovered in modified MSF selective medium [36], with peptone 15 g·L^−1^, potassium phosphate 1 g·L^−1^, magnesium sulfate 0.5 g·L^−1^, granulated agar 20 g·L^−1^, penicillin 0.05 g·L^−1^, and streptomycin 0.3 g·L^−1^ added after autoclaving. Identification of the isolates was done using the PCR technique. Genomic DNA was extracted from pure cultures using the DNeasy Plant Mini Kit (Qiagen, Germany). To perform the PCR, four pairs of specific primers (Uni, Sp13, Sp23 and Sprl) were used to differentiate *Fol* and *Forl* from other special forms of *F. oxysporum* [37]. The amplification reactions were prepared using a final volume of 22 μL. The methodology followed the specifications of the SapphireAmp^®^ Fast PCR Master Mix (Takara, Japan). One µL of DNA template was used in each reaction. All of the reactions were done using a thermocycler (Bioer, China) with the following conditions: 94 °C, 1 min (initial denaturation), followed by 35 cycles that included denaturation at 98 °C for 5 s, alignment at 55 °C for 5 s, extension at 72 for 5 s, and a final elongation at 72 °C for 1 min. The products of the PCR were visualized through electrophoresis in a 1% agarose gel in a TAE buffer (Winkler, Chile) stained with red gel (Biotium, USA), and visualized on a UV transilluminator (Vilber Lourmat, France). The isolates were kept in an MSF medium at 23 °C for subsequent analysis. This analysis was performed two times.

### 2.2. Nanoemulsion Preparation

The preparation of the nanoemulsions was performed by combining high-energy methods based on sonication and high-pressure homogenization. The choice of formulation components was obtained by studying the compatibilities of the extracts with different oils and subsequently, with different surfactants and co-surfactants. Based on the content of saturated fatty acids, the oils used were: coconut oil, palm oil, peanut oil and soybean oil. For the choice of surfactant, the hydrophilic-lipophilic balance (HLB) system was considered for obtaining o/w emulsions, where Polyoxyethylene(10) octyl phenyl ether (Triton X-100) was included in parallel studies, Polyoxyethylene(8) octyl phenyl ether (Triton X-114), polyoxyethylene sorbitan laurate (Tween 20), polyoxyethylene sorbitan palmitate (Tween 40) and polyoxyethylene sorbitan stearate (Tween 80).

Briefly, extract and oils were mixed in equal parts and stirred at room temperature for 48 h. The mixtures were then centrifuged and filtered. The oily component was mixed with the aqueous solution containing 1%, 3% and 5% surfactant and the result of these mixtures was sonicated (Vibra cell^TM^) for 10 min and then subjected to the high pressure homogenizer (EmlsiFlex-C3, Avestin Inc) for 2 h. The obtained formulation was evaluated for transmittance by turbidimetry and the particle size was determined by Dynamic light scattering (DLS) using the Zetasizer Nano ZS system, Malvern. The stability of the nanoemulsion was determined by measuring the particle size variation after three heating-cooling cycles (4 °C–40 °C). For all analyses, the coconut oil extract/Triton X-100 5% (1:99) mixture was found to be the most stable, with particle sizes of 230 nm. 

#### In Vitro Evaluation of Plant Extracts 

The *Fusarium* spores used in the in vitro tests were recovered from the MSF cultures using a sterile loop handle, deposited in a test tube with sterile water, and then the concentration of conidia was measured in a Neabauer chamber in order to perform a spore count under microscopy. After counting, dilutions with sterilized distilled water (SDW) were done in order to obtain 1 × 10^6^ spores·mL^−1^. The extracts from plants of the genera *Psoralea* and *Escallonia* that were used for the in vitro tests were provided by Dr. Iván Montenegro. The concentrations used were 200, 175, 150, 125, 100, 75, 50, 25, 12.5, 6.12 and 3.11 µg·mL^−1^, which were obtained using a stock solution of 5 g of plant extract in 100 µL of DMSO. The 3 mL microplates were then filled with 1200 µL of MSF selective medium mixture, with the extracts added at the range of concentrations indicated above, plus 150 µL of pathogen inoculum [1 × 10^6^ spores·mL^−1^].

The microplates were placed on a shaker at 130 rpm and spore counting was done in a Neubauer chamber after 24 h of incubation at room temperature. This test was repeated two times.

### 2.3. Tomato Plant Trials

These experiments were done in the greenhouse areas of the LF-PUCV. The tomatoes were grown in a heated greenhouse, at 25 °C ± 3 °C. The variety used was Naomi, from seeds provided by the Semillas Latinoamericanas S.A. company. The seedlings were started in 50 cell speedling trays and grown for 21 days (72 mL volume) until being transplanted into 5 L pots with substrate composed of coconut fiber and black peat (Kekkilä profesional, Europe) (1:1). 

For fertigation of the plants, the nutritive solution [38] was used, consisting of the following macronutrients in mmol·L^−1^: 1.25 NH_4_^+^, 8.75 K^+^, 4.25 Ca^2+^, 2.0 Mg^2+^, 13.75 NO_3_^-^, 1.25 H_2_PO_4_^-^ and 3.75 SO_4_^2−^, with a pH of 5.8, and an EC of 2.3 dS·m^−1^, delivered through an emitter of 2 L·h^−1^ in an open system, maintaining 30% humidity. 

When the plants reached a height of 100 cm, treatments of 100 mL of nanoemulsion from plant extracts, at concentrations of 125, 250 and 500 ppm, were applied to the areas surrounding the plant stems. Three days after treatment, 100 mL of inoculum [1·10^6^ spores·mL^−1^] was added per plant. In addition, a chemical treatment was done using dazomet at the dosage recommended by the distributor (35 g·m^−2^). A non-inoculated control treatment (negative control) with the pathogen (T00) and a treatment with water plus inoculum (T0), were also done, applying the pathogens in the manner described above.

### 2.4. Statistical Analysis 

Four blocks, six treatments and six plants were used as the experimental units. The variables evaluated were height of the plant and length of the necrotic streaks in the xylem. 

This experiment was done using a Randomized Complete Block Design (RCBD), to which an Analysis of Variance (ANOVA, *p* ≤ 0.05) was applied, followed by a Tukey’s Test for means separation, using the statistical program GraphPad Prism 8.0.1. 

## 3. Results

### 3.1. Identification of Isolates

**Figure 1:**
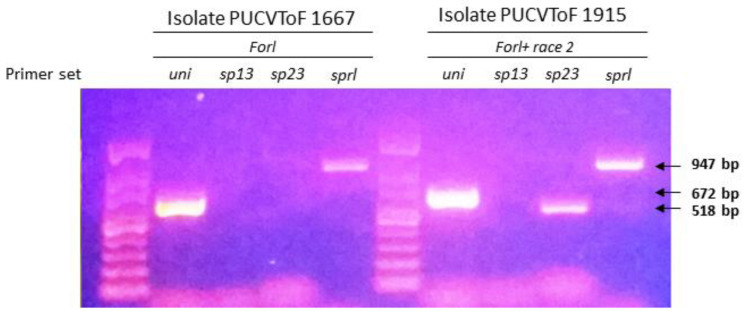
Identification of isolates PUCVToF 1667 and PUCVToF 1915 by polymerase chain reaction (PCR) with the uni, sp13, sp23, and sprl primer sets [37]. PCR products were electrophoresed in a 1.0% agarose gel and stained with red gel. The arrows show the size of the amplicons obtained.

The fungal isolates PUCVToF 1667 and PUCVToF 1915, were obtained from the LF-PUCV fungal collection, and identified using the methodology of Hirano and Arie [37]. For the isolate PUCVToF 1667, PCR products were obtained with the specific primers Uni and Sp23, therefore it would correspond to *Forl*. For the isolate PUCVToF 1915, PCR products were obtained with the specific primers Uni, Spl12 and Sp23, which corresponds to *Forl*, plus an additional band that could correspond to race 2 of *Fol* (Table 1; Figure 1).

### 3.2. In Vitro Evaluation of Plant Extracts on Fusarium Oxysporum Spore Germination 

**Figure 2:**
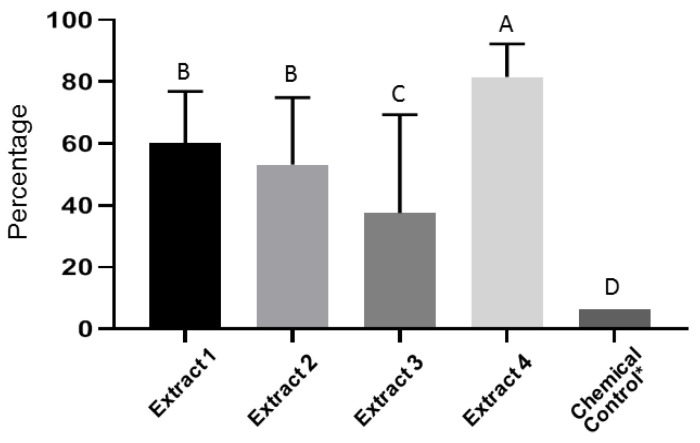
Effect of plant extracts on *Forl* spore germination. The highest percentage of inhibition was observed in the treatment with extract 4 at 200 g·mL^−1^. Means with different letters indicate significant differences from each other (*p* ≤ 0.05).

From the in vitro trials of the *Psoralea* and *Escallonia* genera plant extracts mixed with dichloro and ethyl acetate, arranged on microplates with MSF medium in decreasing concentrations from 200 to 75 g·mL^−1^, it was determined that extract 4 showed reduced spore viability and a greater control of conidia. Greater control (>80 %) was observed for extract 4 at concentrations of 200 g.mL^−1^ (Figure 2). 

### 3.3. Greenhouse Trials

**Figure 3:**
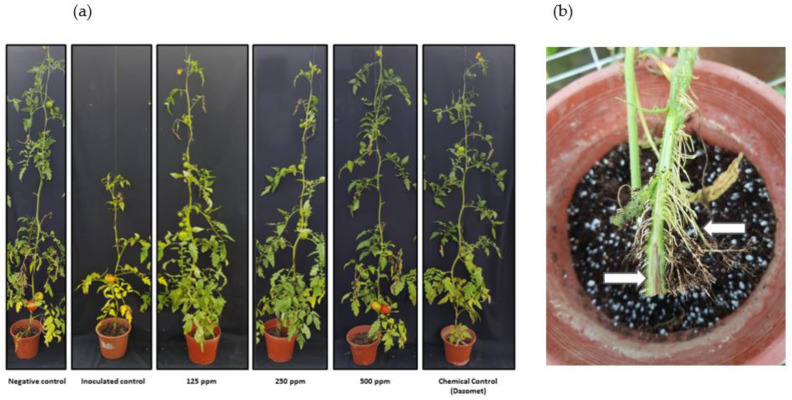
(**a**) Tomato plants cv. Naomi affected by *Forl*. The effects of three concentrations of nanoemulsion (125 ppm, 250 ppm, and 500 ppm), in comparison with two controls, one inoculated with *Forl*, and an uninoculated, negative control, plus a conventional chemical treatment (dazomet). (**b**) Detail of vascular browning in the xylem tissue and abundant root proliferation.

In relation to results from the two greenhouse trials (where the two *Forl* isolates were independently inoculated), the occurrence of disease development attributable to *Fusarium* was observed. The affected plants in both trials showed dark brown vascular lesions around the crown and main roots [39], basal leaves infected with severe chlorosis, and production of adventitious roots (Figure 3). The trials in pots with decreasing nanoemulsion treatment concentrations of 500, 250 and 125 ppm, showed effects on the advance of the pathogen in comparison to the inoculated control treatment (Figure 3).

**Figure 4:**
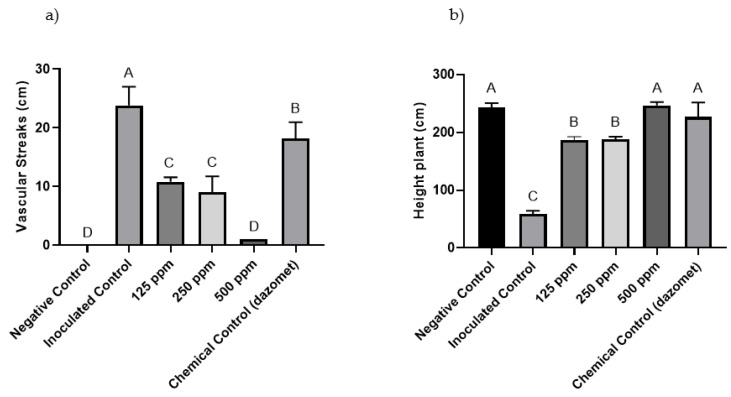
(**a**) Effect of the different concentrations of nanoemulsion (ppm) on the length (cm) of the vascular streaks observed in ‘Naomi’ tomatoes inoculated with *Forl* (PUCVToF 1915). (**b**) Effect of the different concentrations of nanoemulsion (ppm) on plant height (cm) of ‘Naomi’ tomatoes inoculated with *Forl* (PUCVToF 1915). Means with different letters indicate significant differences from each other (*p* ≤ 0.05).

For the plants inoculated with *Forl* + 2 (PUCVToF 1915) and treated with 500 ppm of the nanoemulsion, decreased necrotic lesions were observed, which were statistically equal to the negative control (Figure 4a). The 125 and 250 ppm treatments also decreased the advance of the pathogen, but to a lesser extent than the 500 ppm treatment. With regard to plant height, all the treatments showed a decrease in plant size, except the 500 ppm nanoemulsion and chemical control treatments (Figure 4b). 

**Figure 5:**
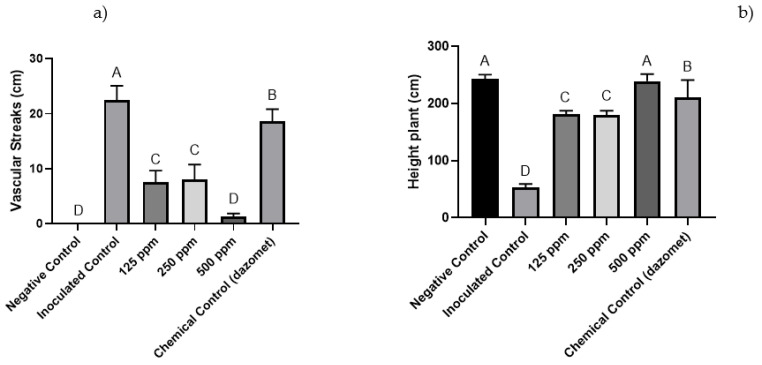
(**a**) Effect of the different concentrations of nanoemulsion (ppm) on the length (cm) of the vascular streaks observed in ‘Naomi’ tomatoes inoculated with *Forl* (PUCVToF 1667). (**b**) Effect of the different concentrations of nanoemulsion (ppm) on plant height (cm) of ‘Naomi’ tomatoes inoculated with *Forl* (PUCVToF 1667). Means with different letters indicate significant differences from each other (*p* ≤ 0.05).

The plants inoculated with *Forl* (PUCVToF 1667) with the 500 ppm treatment, showed a decrease in the advance of the disease, being statistically equal to the negative control treatment (Figure 5a). The plants with 125 and 250 ppm nanoemulsion treatments also showed a decrease in the advance of the pathogen in comparison to untreated, inoculated control plants. With regard to plant height, all the treatments showed a decrease in the size of the plants, with the exception of the 500 ppm nanoemulsion treatment (Figure 5b). 

## 4. Discussion

The fungal isolates obtained from the LF-PUCV showed results consistent with the *Forl* race plus a new or mixture of races between *Fol* + 2 and *Forl* which has an additional band (Table 1, Figure 1), and has the same capacity to produce damage, which is contrary to the expected result as explained by Hirano and Arie [37]. However, both strains were pathogenic and showed similar behaviors in terms of symptoms and effects in the different treatments, therefore both should be considered as *Forl* isolates.

Plant extracts have great potential given their various origins and capacities for inhibiting mycelial growth, spore germination and control over viability of conidia. Nanoemulsions formulated from these plant extracts can be more efficient in controlling pathogens than control with conventional chemicals.

Plant extracts can be used in aqueous or powdered forms [40], or used along with solvents to obtain different compounds, depending on their polarity [41,42]. Among their relatively low-cost advantages, they are also more environmentally friendly and less antagonistic to human health [9]. 

Researchers evaluating the potential phytotoxicity of plant extracts have shown that systemic application has an effect against *Fol* [27]. A study with extracts from plant leaves in the Asteraceae family demonstrated control of *Fol* using these plant extracts, which were found to be 90% effective in vitro, when compared with a reference agrochemical [43]. This is in accordance with the results obtained in this study.

López-Benítez et al. [27], verified inhibition of *Fusarium oxysporum* in vitro at 92% using extracts from *Cinnamomum zeylanicum*, and at 100% using *Larrea tridentata* extract, similar to results obtained by Pérez-Leal et al. [9] using extracts from garlic with 95% inhibition, and in this study, using plant extract 4, based on *Psoralea* and *Escallonia* plants, demonstrating over 80% inhibition.

According to Bowers and Locke [44], extracts from *Piper nigrum*, *Cassia angustifolia* and *Syzygium aromaticum* suppress the development of disease by 80%, which is similar to the results obtained using the *Psoralea* and *Escallonia* extracts in this study. Sharma et al. [32] used nanoemulsions from plant extracts for in vivo studies on controlling *Fol*, and found disease severity was reduced by up to 70%, a value close to the 73% reduction achieved in this study. This is also similar to the garlic extract (var. Rio Sonora) which provides tolerance to *Fol* [11].

Another study using a nanoemulsion based on *Opuntia joconostle*, at a concentration of 150 µL, demonstrated an increased percentage of inhibition, at 68% [33], while still being lower than the 73% observed in this study. In turn, nanoemulsions at concentrations of 3000 mg·L^−1^ reached over 90% inhibition, reaching up to 100% with increasing concentrations [32].

Sharma et al., [32] also recorded greater control of the disease with the use of carbendazim (1000 mg·L^−1^) compared to the 57% obtained in this trial using dazomet (35 g·^m−2^). Although this decreases damage to the plants from *Forl*, the recommended dosage of the fungicide implies higher risks of damage to the environment and to the applicator, as well as a higher cost per hectare. 

## 5. Conclusions

This study is the first report using nanoemulsions of native plant extracts affecting *Fusarium oxysporum* f.sp. *radicis*-*lycopersici* (Forl). Plant extract 4, based on a plant of the genus Psoralea, influenced the in vitro control of *Forl* conidia, reducing their viability compared to the other extracts evaluated. The nanoemulsion formulated based on this extract effectively controlled fusariosis in tomatoes caused by Forl under greenhouse conditions, reducing the damage to the plants, compared to the inoculated control plants. An increase in the concentration of the extracts leads to greater control of the disease.

These results may indicate that the nanoemulsion from plants of the Fabaceae family could be considered a new biological control product for the future. However, these nanoemulsions must be tested under field conditions to verify the biocontrol ability under agricultural soils. Furthermore, this study should be expanded to identify the method of action this nanoemulsion has on the pathogen.

## 6. Patents

Nanoemulsions based on plant extracts were entered into the INAPI registry for intellectual protection of this technology. Chilean Patent Application No. 202003396. 

## Figures and Tables

**Table 1:** Identification of the Isolates. Bands from the PCR amplification of four specific primers for differentiation of the *Fol* and *Forl* races, according to the methodology of Hirano and Arie [37]. Analysis performed twice. ^1^ (+): Presence of amplification; (−): Absence of amplification.

Primer Set	Uni	Sp13	Sp23	Sprl	Result
PUCVToF 1667	+ ^1^	−	−	+	Forl
PUCVToF 1915	+	−	+	+	Forl + race 2

## Data Availability

The data presented in this study are available on request from the corresponding author.
